# Respiratory symptoms and lung function among inmates in a Nigerian prison: a cross sectional study

**DOI:** 10.1186/s12890-022-01882-7

**Published:** 2022-03-14

**Authors:** Joy Nkiru Eze, Obianuju Beatrice Ozoh, Fred Chibuisi Otuu, Elvis Neba Shu, Bond Ugochukwu Anyaehie

**Affiliations:** 1 Department of Paediatrics, College of Medicine, University of Nigeria/University of Nigeria Teaching Hospital, Ituku-Ozalla, Enugu, 400001 Nigeria; 2 Department of Medicine, College of Medicine, University of Lagos, Idi-Araba, Lagos, Nigeria; 3 Department of Pharmaceutics, University of Nigeria, Nsukka, Nigeria; 4 Department of Pharmacology and Therapeutics, College of Medicine, University of Nigeria, Enugu Campus, Enugu, Nigeria; 5 Department of Human Physiology, Faculty of Basic Medical Sciences, College of Medicine, University of Nigeria, Enugu Campus, Enugu, Nigeria

**Keywords:** Respiratory symptoms, Lung function, Prison inmates, Nigeria

## Abstract

**Background:**

Prisoners in low- and middle-income countries are vulnerable to poor lung health from multiple adverse conditions confronted within the prison such as overcrowding, poor ventilation and exposure to second hand smoke. Evidence for poor lung health in this disadvantaged group is needed to inform policy on prison conditions in this region. We assessed the respiratory symptoms and lung function measured by spirometry among prisoners in Enugu, Nigeria and explored the associations between them.

**Methods:**

This was a cross‑sectional study among prison inmates aged 16–76 years. We assessed frequency of respiratory symptoms in the preceding one-year, previous respiratory diagnosis, tobacco smoking status and spirometry. The relationships between respiratory symptoms, smoking status and spirometry pattern were determined using the Chi‑square test.

**Results:**

Of 245 participants, 170 (69.4%) reported at least one respiratory symptom. In all, 214 (87.3%) performed spirometry and 173 (80.8%) had good quality spirometry tests. Using the Global Lung Function Initiative (GLI) predicted values for ‘African Americans’, spirometry results were abnormal in 41 (23.7%) of the participants and when the GLI reference values for ‘Other’ ethnic groups was applied, 78 (45.1%) had abnormal results. Restrictive impairment was most common occurring in 21 (12.1%) and 59 (34.1%) respectively based on the two reference values, and obstructive pattern was found in 18 (10.4%) and 13 (7.5%) respectively. There was no significant association between abnormal spirometry pattern and presence of respiratory symptoms or smoking status. No previous diagnosis for asthma, or bronchitis/chronic obstructive pulmonary disease (COPD) had been made in any of the participants.

**Conclusions:**

We reported high rates of respiratory symptoms and abnormal lung function with under-diagnosis of chronic respiratory diseases among inmates in Enugu prison. The restrictive abnormalities based on GLI equations remain unexplained. There is need for improvement in prison facilities that promote lung health and enhanced access to diagnosis and treatment of respiratory non-communicable disease.

**Supplementary Information:**

The online version contains supplementary material available at 10.1186/s12890-022-01882-7.

## Introduction

Prison inmates are at high risk of disease from multiple colliding adverse conditions that exist in prisons. Overcrowding, inadequate ventilation, indoor air pollution, malnutrition and limited access to preventive and curative healthcare bode for poor health [1–4]. The risks are worse for prisoners in low- and middle-income (LMIC) countries where prison conditions are often more deplorable and many prisoners already face adverse health impacts of poverty.

Prison conditions have been reported to increase the risk of respiratory diseases such as tuberculosis (TB) [3, 5, 5, 6], but there is limited data on the burden of non-communicable respiratory diseases among inmates. Poor indoor air quality in prisons has been documented, and this potentially increases the risk of NCD such as asthma, chronic obstructive pulmonary disease, lung cancer and low lung function [2, 7–9]. High frequency of indoor tobacco smoking among inmates coupled with poor ventilation and limited opportunities for outdoor activities also potentially increase the risk of exposure to indoor air pollution and its adverse health outcomes [2, 8, 10].

Poor air quality has been reported in the Nigerian prison in which this present study is situated, with very high levels (71.03 ± 41.09 μg/m^3^, 89.00 ± 49.66 μg/m^3^, and 107.40 ± 61.86 μg/m^3^) of particulate matters of aerodynamic diameter 1 µm (PM_1_), 2.5 µm (PM_2.5_) and 10 µm (PM_10_) respectively [2]. Levels of total volatile organic compounds (TOVCs) 0.79 ± 0.37 mg/m3 and formaldehyde (HCHO) 0.20 ± 0.07 mg/m^3^ were also higher than maximum allowable air concentration standards, especially in the highly congested cells where inmates smoked tobacco [2]. Increased indoor levels of other air pollutants such as fungal spores which could also lead to respiratory disease have also been reported in other prisons in the United States [11].

There are limited reports of respiratory symptoms among inmates which could be linked to poor indoor air quality. In a Nigerian prison, frequent cough and catarrh was reported in nearly 70% of the inmates and there are currently no reports of lung function measures among inmates from Nigeria [3]. However, spirometry diagnosed chronic obstructive pulmonary disease (COPD) was reported in up to 20% of inmates in Bolvadin closed and open prison, in Turkey [7]. To drive policy for improvement in prison conditions, substantial evidence of adverse impact of the harsh conditions is needed. This need is even more profound in a LMIC such as Nigeria where the inmates are exposed to overcrowded and polluted indoor spaces with limited access to healthcare services [2, 6, 12].

In this study, we aimed to assess the prevalence of respiratory symptoms, and lung function measured by spirometry among inmates in a Nigerian prison with previously documented high levels of poor indoor air quality. We also explored the association between lung function, respiratory symptoms and socio-demographic characteristics.

## Methods

### Study area

We conducted a cross-sectional study from October, 2019 to February, 2020 among inmates at Enugu prison. Enugu prison is a maximum-security prison built 102 years ago. It is one of the oldest prisons in Nigeria and was originally designed for 638 inmates but currently holds 2044 inmates. Enugu is the administrative capital of Enugu State, South-Eastern Nigeria with a population of 722,000 [13], and density estimate of 6,400/km^2^. The prison is located within the metropolis around the major hub of economic and commercial activities.

### Study participants

Study population consisted of adolescent and adult men and women aged 16–76 years old who were crime suspects (awaiting trial) or convicts at the prison. We included participants who had been inmates for a least 1 year and provided informed consent to participate. We excluded those who had relative or absolute contraindications to spirometry including current respiratory infection such as tuberculosis, decompensated cardiac or other chronic disease, kyphoscoliosis, and recent surgery within 3 weeks. We also excluded mentally ill inmates. We employed the consecutive sampling method in the index study. Participants were consecutively recruited from each of the cells until the estimated sample size was achieved.

### Sample size estimation

In a finite population of 2044 prison inmates, where the outcome variable is the proportion of inmates with abnormal spirometry, the following parameters were used for sample size calculation: Proportion with spirometry-diagnosed COPD from a previous study [7], estimated at (20%); at 95% of confidence level, and 5% accuracy level. A sample size of 221 was calculated but we recruited 245 inmates to accommodate for an expected 10% who may not perform good quality spirometry.

### Data collection

#### Questionnaire administration

A trained interviewer used a standard proforma to obtain socio-demographic information during a face to face encounter within the prison facility but outside the cell. Information obtained included the age, gender, prison status (e.g. awaiting trial, condemned, or asylum), and duration of incarceration. We used a modified version of the validated International Multidisciplinary Programme to Address Lung Health and TB in Africa (IMPALA) respiratory symptom and life exposure questionnaires to obtain information on respiratory symptoms (occurrence, type and frequency of respiratory symptoms in the previous one year), smoking history and exposure to air pollution, respectively [14]. The IMPALA questionnaires were developed by the strategic multi-disciplinary partnership of paediatric and adult lung health investigators from Benin, Cameroon, Ethiopia, Ghana, Kenya, Malawi, Nigeria, South Africa, Sudan, Tanzania and Uganda in partnership with lay in-country representatives following a rigorous process out of a perceived need for a validated context-sensitive tool tailored to the African context for accurately estimating the prevalence and risk factors of NCD. These questionnaires have been previously validated following initial pilot studies in Malawi, and were found reliable for assessing respiratory symptoms and exposures among Africans. Although the questionnaires were designed for assessing the impact of indoor air pollution in the home environment from cooking, heating and lighting sources; and life time exposure to outdoor smoke and fumes on respiratory disease burden, we modified it for the prison study by excluding aspects such as use of cooking fuels and lighting sources that did not apply to the situation in the prison.

The impala questionnaires are written in English which is the official language of communication in Nigeria and most people in Nigeria can speak English at least the ‘broken English’ otherwise known as ‘pidgin English’. The questionnaires were administered to all participants by one of the investigators who paraphrased the questions in a consistent manner for those who did not understand standard English. The respiratory symptoms that were assessed included cough, catarrh, phlegm, shortness of breath, wheeze, and haemoptysis. History of weight loss, and previous diagnosis of tuberculosis, bronchitis/COPD, asthma, allergic rhinitis, and heart disease were obtained. Exposure to indoor and outdoor smoke, and smoking status was documented.

We categorized the symptoms on the ‘IMPALA’ questionnaire into:

*‘Cough’:* recurrent morning, daytime or night cough during cold weather (with increased severity if it occurred on most days for as long as three months each year).

*‘Phlegm’:* recurrent morning, daytime or nighttime sputum production during cold weather (or sputum that is difficult to bring up when you don't have a cold) with increased severity if it occurred on most days for as long as three months each year.

*‘Shortness of Breath* (SOB)’: breathlessness on leaving the room, on dressing or undressing, or when hurrying on level ground or a slight hill (with increasing severity based on additional symptoms such as getting short of breath when walking with other people of same age or if has to stop for breath when walking at own pace).

*‘Wheeze’*: whistling in the chest in the past 12 months triggered by a dusty or smoky environment (with increasing severity as to limit speech to only one or two words a time between breaths).

*‘Night sweats’*: sweating more than usual for the heat at night in the last 4 weeks.

*‘Catarrh’*: was defined as mucus accumulation in the nose, throat or sinuses. It is also referred to as ‘post nasal drip’

*‘Any Respiratory Symptom’:* presence of any of the above respiratory symptoms.

*‘Previous respiratory diagnosis’*: Diagnosis made by a doctor or other health care provider for any of the following disease conditions- tuberculosis, Bronchitis/COPD, asthma, and allergic rhinitis, and heart disease.

We categorized their smoking status as follows [15]:

‘Current smokers’: defined as those who smoked for at least 1 month and who continued to smoke.

‘Former smokers’: defined as those who had quit smoking at least one month before the study.

‘Non-smokers’: those who never smoked in their life-time.

We also categorized other exposures as follows:

Non-Occupational: breathing in vapours, dusts, gases or fumes for more than 15 h per week while in or outside the cell.

‘Exposure to aerosols or sprays within the prison cell’: breathing in insecticide, deodorant, cleaning spray and any other aerosol within the prison cell.

‘Exposure to refuse burn’: breathing in of smoke from burning of refuse (waste and rubbish) outside the cell or prison environment.

Anthropometric measurements.

Height and weight were measured using the standard protocols recommended by the World Health Organisation (WHO) [16]; and the body mass index (BMI) was calculated using the formula: Weight (Kg)/square of Height in meters (m^2^).

#### Spirometry

Lung function parameters: FEV_1_, FVC, FEV_1_/FVC ratio (FEV_1_%), were measured on eligible participants using the ndd *EasyOne*® spirometer (ndd Medical Technologies, Andover, Massachusetts, USA), following the American Thoracic Society and European Respiratory Society (ATS/ ERS) guidelines [17, 18]. The spirometry procedure was performed in the prison clinic area by one of the investigators who holds a Pan African Thoracic Society (PATS) certificate of competence in spirometry. A minimum of three and a maximum of eight FVC maneuvers were performed by each participant; and the best trial of three acceptable and repeatable tests was recorded.All tests results were individually checked and independently verified for quality according to the ATS/ERS acceptability, repeatability, and usability criteria. Only acceptable pre-bronchodilator tests were included for analysis. Standard measures for infection prevention and control were strictly adhered to during the spirometry procedure through handwashing and use of alcohol-based hand sanitizers (containing at least 70% alcohol) by each participant and the investigator before contact. Disposable, in-line filters and mouth pieces were also used for each participant and handled by the participants after use. The tests were conducted prior to the COVID-19 pandemic so additional protection such as face masks and shields were not used. All disposable items were carefully disposed of at the end of each testing session and spirometer wiped down.

We utilized the *Global Lung Function Initiative* (GLI) reference equation for ‘African Americans’ to determine spirometry pattern [19]. Lung function abnormalities were defined using the Lambda-mu-sigma (LMS) derived lower limit of normal (LLN_5_) of zFEV_1_ /FVC at -1.64 (21). We also compared frequencies of spirometry patterns using the African American reference with results obtained using reference values for ‘Other’ ethnic group for robustness of results.

The results of the spirometry test were interpreted as follows [20]:

Obstructive ventilatory pattern—FEV_1_/FVC < LLN; with FVC ≥ LLN

Restrictive ventilatory pattern—FEV_1_/FVC ≥ LLN; with FVC < LLN and

Mixed obstructive-restrictive pattern- FEV_1_/FVC < LLN with FVC < LLN

Normal- FEV_1_/FVC ≥ LLN; with FVC ≥ LLN.

The presence of any of the abnormal patterns was considered as *‘Abnormal Spirometry’*.

The severity of impairment based on FEV_1_ and FVC respectively was further categorized using the z-scores as proposed by Quanjer et al. [22] as follows:

Mild: z-score ≥ -2

Moderate: -2.5 to < -2

Moderately severe: -3 to < -2.

Severe: -4 to < -3.

Very severe: z-score < -4.

### Statistical analysis

The Kolmogorov–Smirnov test was used to determine the normality of variables. Discrete variables (clinical symptoms, lung function impairment and smoking status) were presented as frequencies, and percentages. Continuous variables (lung function values, weight, height, BMI) were expressed as means ± standard deviation. Variables that assumed non-Gaussian distribution (age) was expressed as median and interquartile range. Chi-square test or Fisher’s exact test were applicable was used to for difference between in relation to smoking status, presence and frequency of respiratory symptoms, and abnormal spirometry pattern as well as to test for association between variable (smoking status, presence of respiratory symptoms, and lung function abnormality). Statistical analyses were performed using the statistical package for the social sciences (SPSS) version 21 (IBM SPSS Inc., Chicago, IL, USA). Statistical significance was set at *p* < 0.05.

## Results

**Fig. 1:**
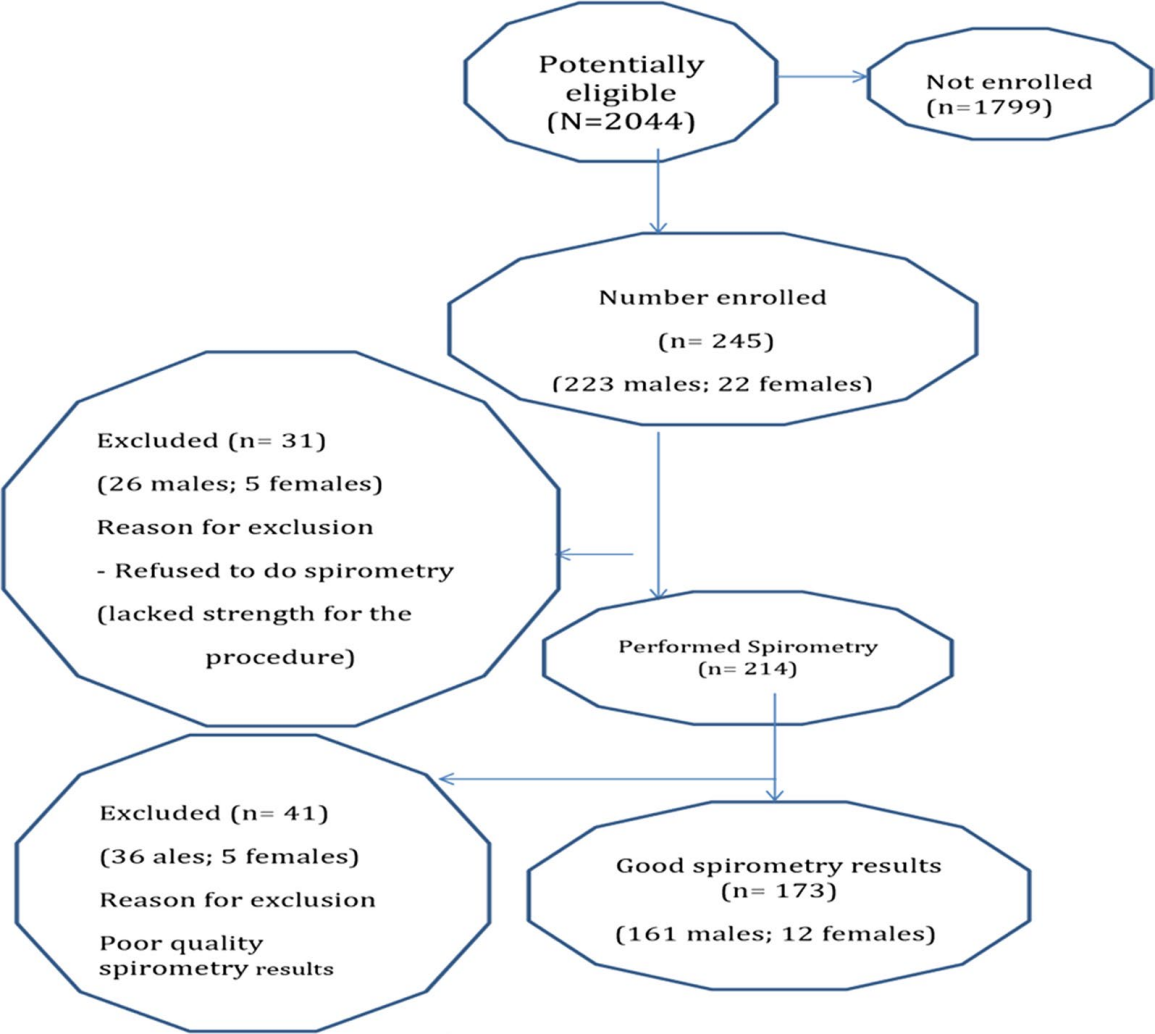
Screening and enrolment of participants

The prison had a total population of 2044 inmates (1998 males and 46 females). Only, 245 participants (223 males and 22 females) were enrolled for the study; male: female ratio 10.1:1. Of these, 214 (197 males and 17 females) performed spirometry giving a completion rate of 87.3%. The process of enrolment is depicted in Fig. 1.

### Participant’s characteristics

The basic characteristics of the 245 study participants are shown Table 1. The participants were aged 16 years to 76 years old; median age was 29 (IQR 60) years. Majority of them 164 (66.9%) had normal body mass index (BMI), and 58 (23.7%) were overweight; mean BMI (SD) = 23.6 (3.7) kg/m^2^.

**Table 1:** Socio-demographic characteristics of 245 participants

Characteristic	All participants N = 245 n (%)	Male N = 223 n (%)	Female N = 22 n (%)
Median age (IQR) in years	29 (60)	29 (60)	31.5 (39)
*Age group*			
16–25	76 (31.0)	71 (31.8)	5 (22.7)
36–45	98 (40.0)	88 (39.5)	10 (45.5)
46–55	42 (17.1)	40 (17.9)	2 (9.1)
56–65	21 (8.6)	16 (7.2)	5 (22.7)
> 65	8 (3.3)	8 (3.6)	0 (0)
Mean ± SD BMI in kg/m^2^	23.6 ± 3.7	23.5 ± 3.5	25.0 ± 5.1
*BMI Category ( kg/m*^*2*^*)*			
Underweight (< 18.5)	7 (2.9)	5 (2.2)	2 (9.1)
Normal (≥ 18.5 and < 25)	164 (66.9)	156 (70.0)	8 (36.4)
Overweight (25–29.9)	58 (23.7)	49 (22.0)	9 (40.9)
Obesity > 30	16 (6.5)	13 (5.8)	3 (13.6)
Mean ± SD duration of stay (months)	31.3 ± 18.2	32.3 ± 18.2	20.8 ± 14.3
*Smoking status*			
Current tobacco smoker	158 (64.5)	155 (69.5)	3 (13.6)
Former smoker	22 (9.0)	22 (9.9)	0 (0)
None smoker	65 (26.5)	46 (20.6)	19 (66.4)
*Respiratory symptoms present*			
Yes	170 (69.4)	155 (69.5)	15 (68.2)
No	75 (30.6)	68 (30.5)	7 (31.8)
*Frequency of respiratory symptoms*			
1–2 per year	138 (81.2)	126 (81.3)	12 (80.0)
3–6 per year	14 (8.2)	14 (9.0)	0 (0)
> 6 per year	18 (10.6)	15 (9.7)	3 (20)

BMI = Body Mass Index; SD = Standard deviation

The mean (SD) duration of incarceration was 31.3 (18.2) months. In all, 158 (64.5%) were current tobacco smokers; 22 (9.0%) were former smokers; 65 (26.5%) had no smoking history. However, all participants were exposed to passive smoke as those who smoked did so inside the cells.

### Respiratory symptoms

Among 245 inmates, 170 (69.4%) had *‘Any Respiratory Symptom’*. The reported symptoms (in descending order of frequency) were: *‘Cough’* 170 (69.4%), ‘*Catarrh’* 170 (69.4%), *‘Phlegm’* 166 (67.8%), *‘SOB’* 2 (0.8%) during the day or night in cold weather respectively.

One hundred and thirty eighty (81.2%) of those who experienced any respiratory symptoms had symptoms about once or twice per year; 14 (8.2%) and 18 (10.6%) respectively had symptoms three to six times per year and more than six times per year, each lasting between one to two weeks, Table 1.

The basic characteristics of the participants with good and poor quality spirometry respectively were also compared; Supplementary Table 1. The frequency of respiratory symptoms as well as smoking habits did not differ between those with good quality spirometry and those with poor quality.

### Frequency of respiratory symptoms in relation to smoking status

Smokers 115/158 (72.8%) had more respiratory symptoms compared to non-smokers 41/65 (63.1%), Table 2. There was no significant association between smoking status and occurrence of respiratory symptoms in males, *p* = 0.494; and females, *p* = 0.752.

**Table 2:** Frequency of Respiratory Symptoms in relation to Smoking Status among 245 participants

Gender	Smoking status	None n (%)	1–2 n (%)	3–6 n (%)	> 6 n (%)	*p* value*
Male	Current smoker	42 (27.1)	94 (60.6)	10 (6.5)	9 (5.8)	0.494
	Former smoker	8 (36.4)	10 (45.5)	1 (4.5)	3 (13.6)	
	None smoker	18 (39.1)	22 (47.8)	3 (6.5)	3 (6.5)	
Female	Current smoker	1 (33.3)	2 (66.7)	0 (0)	0 (0)	0.752
	Former smoker	0 (0)	0 (0)	0 (0)	0 (0)	
	None smoker	6 (31.6)	10 (52.6)	0 (0)	3 (15.8)	
All participants	Current smoker	43 (27.2)	96 (60.8)	10 (6.3)	9 (5.7)	0.463
	Former smoker	8 (36.4)	10 (45.5)	1 (4.5)	3 (13.6)	
	None smoker	24 (36.9)	32 (49.2)	3 (4.6)	6 (9.2)	

^*^Fisher’s exact test

### Previous respiratory conditions

Sixteen (6.5%) had a history suggestive of allergic rhinitis. One inmate had previous tuberculosis; while one had self-reported cough and wheeze. None of the participants had been diagnosed for bronchitis/chronic obstructive pulmonary disease (COPD).

### Spirometry pattern and severity of impairment

Table 3 shows the mean ± SD of lung function parameters FEV_1_ (3.1 ± 0.7 L); FVC (3.9 ± 0.8 L); and FEV_1_/FVC ratio (80.3 ± 6.6) for 173 participants with acceptable spirometry respectively.

**Table 3:** Mean lung function values in 173 participants

Variable	Mean (SD)					
	FEV_1_ (litres)	zFEV_1_ (litres)	FVC (litres)	zFVC (litres)	FEV_1_/FVC	zFEV_1_/FVC
Males, n = 161	3.2 ± 0.6	-0.8 ± -1.0	4.0 ± 0.7	-0.5 ± 0.9	80.4 ± 6.5	-0.5 ± 0.9
Females, n = 12	2.0 ± 0.4	-1.8 ± 1.0	2.5 ± 0.4	-1.5 ± 1.0	79.2 ± 9.1	-0.7 ± 1.1
All participants, N = 173	3.1 ± 0.7	-0.8 ± 1.0	3.9 ± 0.8	-0.6 ± 1.0	80.3 ± 6.6	-0.5 ± 0.9

We compared the frequencies of spirometry patterns using the GLI ‘African American’ reference values with those of ‘Other’ ethnic groups, Table 4. We observed a significant difference in frequencies of various spirometry patterns.

**Table 4:** Lung function pattern classified by GLI ‘African American’ and ‘Other’ reference values

	GLI reference for African Americans				GLI reference for ‘Other ethnic groups’		
Spirometry pattern		All participants N = 173 (%)	Males n = 161 (%)	Females n = 12 (%)	All participants N = 173 (%)	Male n = 161 (%)	Female n = 12 (%)
Normal		132 (76.3)	126 (78.2)	6 (50.0)	95 (54.9)	92 (57.1)	3 (25.0)
Obstructive		18 (10.4)	15 (9.3)	3 (25)	13 (7.5)	12 (7.5)	1 (8.3)
Restrictive		21 (12.1)	18 (11.2)	3 (25)	59 (34.1)	52 (32.3)	7 (58.3)
Mixed		2 (1.2)	2 (1.2)	0 (0)	6 (3.5)	5 (3.1)	1 (8.3)

Using the African American reference values, spirometry results were normal in 132 (76.3%) of participants while 41 (23.7%) had abnormal spirometry results. When the reference values for ‘Other’ ethnic groups was applied, 95 (54.9%) participants had normal spirometry while 78 (45.1%) had abnormal results.

Restrictive spirometry impairments occurred in 21 (12.1%) and 59 (34.1%) of participants respectively based on reference values for African Americans and ‘Other’ ethnic groups respectively. Obstructive impairment occurred in 18 (10.4%) based on African American values and 13 (7.5%) based on reference values for ‘Other’ ethnic groups.

### Grading of impairment

Based on the zFEV1 score, severity of lung function impairment were classified as follows: mild 18 (10.4%); moderate 11 (6.4%); moderately severe 5 (2.9%); and severe 3 (1.7%) respectively.

When based on the zFVC score, 13 (7.5%), 5 (2.9%), 4 (2.3%), 1 (0.6%) and 1 (0.6%) of participants had mild, moderate, moderately severe, severe and very severe impairment respectively.

### Relationship between spirometry pattern, respiratory symptoms, and smoking status

There was no significant association between spirometry pattern and presence of respiratory symptoms (X^2^ = 4.970, *p* = 0.174), Table 5; nor between smoking status and spirometry patterns (X^2^ = 3.145, *p* = 0.790); Table 6.

**Table 5:** Relationship between spirometry pattern and respiratory symptom

Gender	Spirometry pattern	Respiratory symptom		*p* value*
		Present n (%)	Absent n (%)	
Male	Mixed	2 (100)	0 (0)	
	Obstructive	11 (73.3)	4 (26.7)	0.274
	Restrictive	16 (88.9)	2 (11.1)	
	Normal	87 (69.0)	39 (31.0)	
Female	Mixed	0 (0)	0 (0)	
	Obstructive	2 (66.7)	1 (33.3)	0.513
	Restrictive	3 (100)	0 (0)	
	Normal	4 (66.7)	2 (33.3)	
All participants	Mixed	2 (100)	0 (0)	
	Obstructive	13 (72.2)	5 (27.8)	0.174
	Restrictive	19 (90.5)	2 (9.5)	
	Normal	91 (68.9)	41 (31.1)	

^*^Fischer’s exact test

**Table 6:** Relationship between smoking status and spirometry pattern

Gender	Smoking status	Spirometry pattern				*p* value*
		Mixed n (%)	Obstructive n (%)	Restrictive n (%)	Normal n (%)	
Male	Current smoker	1 (0.9)	11 (9.8)	11 (9.8)	89 (79.5)	0.744
	Former	0 (0)	2 (14.3)	1 (7.1)	11(78.6)	
	None smoker	1 (2.9)	2 (5.7)	6 (17.1)	26 (74.3)	
Female	Current smoker	0 (0)	1 (33.3)	1 (33.3)	1 (33.3)	0.801
	Former	0 (0)	0 (0)	0 (0)	0 (0)	
	None smoker	0 (0)	2 (22.2)	2 (22.2)	5 (55.6)	
All participants	Current smoker	1 (0.9)	12 (10.4)	12 (10.4)	90 (78.3)	
	Former smoker	0 (0)	2 (14.3)	1 (7.1)	11 (78.6)	0.790
	None smoker	1 (2.3)	4 (9.0)	8 (18.2)	31 (70.5)	

^*^Fischer’s exact test

## Discussion

There is growing interest in the lung health of Africans due to recognition of the increasing burden of both indoor and outdoor air pollution in Sub-saharan Africa (SSA) [21–23]. Previous studies have focused on community dwelling individuals and vulnerable groups such as prisoners who face multiple drivers of poor lung health have not been well explored in SSA and indeed globally [7, 21–24].

The main findings from this present study are a relatively high prevalence of spirometry impairment (23.7%) and respiratory symptoms (69.4%) among prison inmates in Enugu. Frequency of restrictive impairment (12.1%) using the recommended African American reference equation was modest. Although this study was not designed to diagnose COPD, 6.4% of participants had moderate obstruction and none had a previous respiratory diagnosis nor on treatment for obstructive lung disease. There was no association between the presence of respiratory symptoms and spirometry pattern, nor between smoking status and spirometry pattern.

Noteworthy is the observed difference in the proportion of spirometry abnormalities using the GLI African American reference standards (23.7%) compared with the ‘Others’ standards (45.1%) to characterize spirometry pattern and quantify abnormal spirometry [25]. Similar substantial disparities were reported by Obaseki et al. [23], in a study of community dwelling adults in Nigeria using different reference standards. They reported a prevalence of reduced FVC of 70.4% for men and 72.8% for women when using NHANES values for Caucasian Americans which were significantly higher than rates of 17.8% for men and 14.4% for women when using NHANES equations for African-American or the GLI equations for ‘black’ persons of 15.5% for men and 20.5% for women. These recognized differences in rates when different reference standards were applied further strengthen the need for inclusion of a reference values for Africans that is derived from a large population of Africans into the GLI equations. However, arguments on the need for racial adjustments for lung function are also emerging and premise on the potential role of socio-economic factors that influence lung growth independent of race [26].

The frequency of abnormal spirometry particularly the obstructive pattern in this present study is similar to those reported previously among inmates (8, 29). However, restrictive impairment is higher than in the general population. Comparison of our findings using the ‘others’ equation with those of young Nigerian adults and adolescent students in a previous study (that also used the ‘others’ reference equation) demonstrates a higher frequency of restrictive patterns (34.1%) among prisoners compared to the community dwelling adults (25.4%) [28]. This suggests that perhaps socio-economic and environmental factors including exposure to recognized risk factors for lung function impairment such as overcrowding, malnutrition, indoor tobacco smoke and mould which are pervasive in many prisons may have a role. Reduced lung function among these prisoners portends increased risk of future adverse outcomes including mortality and therefore calls for improvement in prison facilities and living conditions [24].

The high frequency of respiratory symptom (69.4%) among prisoners in this study is consistent with previous findings (68.7%) by Fred & Elvis in the same Enugu prison in 2019 [3]. The consistency in this finding implies that poor conditions within the prison have persisted or may have worsened and brings to the fore the decadence in prison facilities in Nigeria and the need for advocacy to change this paradigm. Increased frequency of respiratory symptoms among smokers compared to non-smokers that was reported in this present study is plausible and expected and has also been previously reported both in a prison population and in the general population [7, 15, 29]. With more than 60% of the participants in our study currently smoking indoors, it implies that most prisoners are exposed to secondhand smoke and therefore also at risk of lung diseases associated with cigarette smoking.

Striking is that despite the high burden of respiratory symptoms and lung function abnormalities including obstructive impairment among these inmates, no prisoner has a previous respiratory diagnosis nor on treatment for any chronic respiratory disease. It alludes to the reported limited access to healthcare services experienced by inmates in Nigeria leading to poor health outcomes [30, 31]. The lack of association between abnormal spirometry and respiratory symptoms showcases the need to include spirometry as a priority service in the development of interventions to improve healthcare service to inmates in prisons globally.

### Limitations of the study

The first recognized limitation in this study is recall bias for assessing the history of respiratory symptoms as we did not have access to clinical notes. Some of the inmates may not recall accurately the number of episode of respiratory symptoms in the previous one year. Also, a few of the inmates could not recall their actual date of birth, thus age estimation was based on events surrounding the period and year of birth. Use of estimated age may have affected their lung function interpretation. Lastly, the smoking status of prisoners was documented but information on the number of sticks of cigarette smoked per day was not obtained for further sensitivity analysis.

However, this study has provided the lung function parameters of prison inmates in a Nigerian prison which was previously lacking. The reported high rates of abnormal lung function compared to the general population lays credence to the potential adverse impact of poor prison condition on respiratory health. This data is useful for informing policy, driving advocacy and proposing recommendations for reduction in prison congestion, improvement in ventilation as well as access to healthcare for early diagnosis and treatment of respiratory NCDs. Policy towards smoke-free prisons is also recommended to reduce the risk of passive smoking which could improve indoor air quality and perhaps lung health.

## Conclusion

In Conclusion, we have reported high rates of respiratory symptoms and abnormal lung function with under-diagnosis of chronic respiratory diseases among inmates in Enugu prison. This calls for improvement in prison facilities to promote lung health and enhance access to diagnosis and treatment of respiratory NCD among inmates.

## Supplementary Information

**Additional file 1**. **Supplementary Table 1.** Socio-demographic characteristics of 214 participants with and without good spirometry; and IMPALA respiratory symptoms and life exposure questionnaires.

## Data Availability

The data that support the findings of this study are available from the authors upon reasonable request and with permission of the prison authorities.

## Abbreviations

COPD: Chronic obstructive pulmonary disease
GLI: Global Lung Function Initiative
HCHO: Formaldehyde
IMPALA: International Multidisciplinary Programme to Address Lung Health and TB in Africa
LMIC: Low- and middle-income countries
NCD: Non-communicable disease
TOVCs: Total volatile organic compounds

## References

[1] Yordi Aguirre I, Ahalt C, Atabay T, Baybutt M, Van den Bergh B, Chorgoliani D, et al. Prisons and Health. Enggist S, Møller L, Galea G, Udesen C, editors. World Health Organization. Denmark: World Health Organization Regional Office for Europe; 2014. 1–189. https://apps.who.int/iris/bitstream/handle/10665/128603/PrisonandHealth.pdf. Accessed 26 May 2021.

[2] Otuu F, Okwuosa C, Maduka I, Ogbodo S, Shuneba I, Nkechi H (2019). Indoor air quality, cell features and lifestyle characteristics: implications on the prevalence of some respiratory tract diseases and symptoms among inmates of Enugu Prison, Nigeria. Adv Clin Toxicol..

[3] Otuu F, Shu E (2019). Prevalent diseases among inmates in three federal prisons in South-East Geopolitical Zone of Nigeria: A Peep into the Environmental Factors. J Environ Sci Public Heal.

[4] British lung foundation. Briefing : health inequalities and lung disease. 2018;(326730). https://www.blf.org.uk/sites/default/files/British Lung Foundation - Lung disease and health inequalities briefing.pdf. Accessed 14 Jan 2020.

[5] Cernat T, Comanescu M, Alexandru D, Carlig V (2010). Simoultaneuos occurence of other diseases among prison inmates with tuberculosis. Curr Health Sci J.

[6] Dara M, Acosta CD, Melchers NVSV, Al-Darraji HAA, Chorgoliani D, Reyes H (2015). Tuberculosis control in prisons: current situation and research gaps. Int J Infect Dis.

[7] Kuupiel D, Vezi P, Bawontuo V, Osei E, Mashamba-Thompson TP (2020). Tuberculosis active case-finding interventions and approaches for prisoners in sub-Saharan Africa: a systematic scoping review. BMC Infect Dis.

[8] Turan O (2015). Smoking status and the presence of chronic obstructive pulmonary disease in prison. J Addict Med.

[9] Semple S, Sweeting H, Demou E, Logan G, O’Donnell R, Hunt K (2017). Characterising the exposure of prison staff to second-hand tobacco smoke. Ann Work Expo Heal.

[10] Sannier O, Gignon M, Defouilloy C, Hermant A, Manaouil C, Jardé O (2009). Dépistage de l’asthme et de la bronchopneumopathie obstructive à la maison d’arrêt d’Amiens : étude préliminaire transversale. Rev Pneumol Clin.

[11] Semple S, Dobson R, Sweeting H, Brown A, Hunt K (2020). The impact of implementation of a national smoke-free prisons policy on indoor air quality: results from the Tobacco in Prisons study. Tob Control.

[12] Ofungwu J (2005). Indoor air quality investigation and health risk assessment at correctional institutions. Integr Environ Assess Manag.

[13] United Nations. Prison Reform and Alternatives to Imprisonment. https://www.unodc.org/unodc/en/justice-and-prison-reform/prison-reform-and-alternatives-to-imprisonment.html. 26 May 2021.

[14] National Population Commission. Population Distribution by Sex, State,LGA & Senatorial District. 2010;III:1–64. http://www.population.gov.ng/images/Vol 03 Table DSx LGAPop by SDistrict-PDF.pdf. Accessed 20 Aug 2020.

[15] Saleh S, van Zyl-Smit R, Allwood B, Lawin H, Ngahane BHM, Ayakaka I (2018). Questionnaires for lung health in Africa across the life course. Int J Environ Res Public Health.

[16] Isabel U, Alberto C, María QJ, Nerea M, Xavier B, Jordi S (2005). Smoking habit, respiratory symptoms and lung function in young adults. Eur J Public Health.

[17] World Health Organization Expert Committee. Physical Status: The use and Interpretation of Anthropometry, report of a WHO expert committee (1995). World Health Organization https://apps.who.int/iris/handle/10665/37003 8594834

[18] Miller MR, Hankinson J, Brusasco V, Burgos F, Casaburi R, Coates A (2005). Standardisation of spirometry. Eur Respir J.

[19] Graham BL, Steenbruggen I, Barjaktarevic IZ, Cooper BG, Hall GL, Hallstrand TS (2019). Standardization of spirometry 2019 update an official American Thoracic Society and European Respiratory Society technical statement. Am J Respir Crit Care Med.

[20] Quanjer PH, Stocks J, Cole TJ.(2012). Global Lung Function Initiative (GLI) 2012. All-Age Multi-Ethnic Reference Values for Spirometry. Advantages and consequences. https://www.ers-education.org.2012.pdf. Accessed 20 Aug 2020.

[21] Fasola S, La GS, Cibella F, Cilluffo G, Viegi G (2017). Global Lung Function Initiative 2012 reference values for spirometry in South Italian children. Respir Med.

[22] Quanjer PH, Pretto JJ, Brazzale DJ, Boros PW, Quanjer PH (2014). Grading the severity of airways obstruction: new wine in new bottles. Eur Respir J.

[23] Rylance S, Naunje A, Mbalume F, Jewell C, Balmes JR, Grigg J (2019). Lung health and exposure to air pollution in Malawian children (CAPS): a cross-sectional study Paediatric lung disease. Thorax.

[24] Nightingale R, Lesosky M, Flitz G, Rylance SJ, Meghji J, Burney P (2019). Noncommunicable Respiratory Disease and Air Pollution Exposure in Malawi (CAPS). A Cross-Sectional Study. Am J Respir Crit Care Med.

[25] Obaseki DO, Erhabor GE, Awopeju OF, Adewole OO, Adeniyi BO, Buist EAS (2017). Reduced forced vital capacity in an African population prevalence and risk factors. Ann Am Thorac Soc.

[26] Backman H, Eriksson B, Hedman L, Stridsman C, Jansson SA, Sovijärvi A (2016). Restrictive spirometric pattern in the general adult population: Methods of defining the condition and consequences on prevalence. Respir Med.

[27] Eze JN, Ozoh OB, Otuu FC, Shu EN, Anyaehie BU. Respiratory symptoms and lung function among inmates in a Nigerian Prison. In: Proceedings of the American Thoracic Society Conference; 2021 May 14–19; Virtual. Am J Respir Crit Care Med 2021;203:A2175 www.atsjournals.org

[28] Braun L (2014). Breathing race into the machine: the surprising career of the spirometer from plantation to genetics.

[29] Harzke AJ, Baillargeon JG, Pruitt SL, Pulvino JS, Paar DP, Kelley MF (2010). Prevalence of chronic medical conditions among inmates in the texas prison system. J Urban Heal.

[30] Ozoh OB, Eze JN, Adeyeye OO, Eromosele O, Dede SK, Ndukwu CI, et al. Unrecognized Respiratory Morbidity among Adolescents and Young Adults in Nigeria : Implications for Future Health Outcomes. NMJ 2020;22:0–7. 10.4103/nmj.NMJ_36_20 PMC7688032 33284873

[31] Özdemir T, Kasapoğlu B, Akkuş İ, Kaya F, Pirinçci E, Eren S (2020). Analysis of the relationship between smoking and chronic respiratory symptoms, level of income and education. J Contemp Med.

[32] Joshua IA, Dangata YY, Audu O, Nmadu AG, Omole NV (2014). Human rights and Nigerian prisoners—Are prisoners not humans?. Med Law.

[33] Ochonma O, Chijioke O (2019). Healthcare services satisfaction in the prison: a cross-sectional survey and comparative analysis of patient in-mates’ perspectives in an african country. Int Inst Acad Res Dev.

[34] Santelli J, Haerizadeh S, McGovern T. Inclusion with Protection: Obtaining informed consent when conducting research with adolescents. Innocenti Research Briefs 2017- 05, Methods: Conducting Research with Adolescents in Low- and Middle-Income Countries, no. 3, UNICEF Office of Research, Innocenti, Florence, 2017.

