# Supplementary material for: Respiratory symptoms and lung function among inmates in a Nigerian prison: a cross sectional study
Source: BMC Pulm Med. 2022 Mar 14;22:84. doi: 10.1186/s12890-022-01882-7 (PMC8918425; doi:10.1186/s12890-022-01882-7)
Supplement: Supplementary file 1 — Additional file 1. Supplementary Table 1. Socio-demographic characteristics of 214 participants with and without good spirometry; and IMPALA respiratory symptoms and life exposure questionnaires. [file 12890_2022_1882_MOESM1_ESM.docx]

Supplementary Table 1: Socio-demographic characteristics of 214 participants with and without good spirometry

| **Characteristic** | | **All participants**  **N=214**  **n (%)** | **Participants with good quality spirometry**  **N=173**  **n (%)** | | | **Participants with poor quality spirometry**  **N=41**  **n (%)** | **p value** |  |  |
| --- | --- | --- | --- | --- | --- | --- | --- | --- | --- |
| Median age (IQR) in years | | 29 (60) | 29 (60) | | | 29.0 (39) |  |  |  |
| *Age group* | |  |  | | |  |  |  |  |
| 16-25 | | 69 (32.2) | 55 (31.8) | | | 14 (34.1) | 0.597^†^ |  |  |
| 26-35 | | 85 (39.7) | 71 (41.0) | | | 14 (34.1) |  |  |  |
| 36-45 | | 36 (16.8) | 26 (15.0) | | | 10 (24.4) |  |  |  |
| 46-55 | | 18 (8.4) | 15 (8.7) | | | 3 (7.3) |  |  |  |
| 56-65 | | 3 (1.4) | 3 (1.7) | | | 0 (0) |  |  |  |
| >65 | | 3 (1.4) | 3 (1.7) | | | 0 (0) |  |  |  |
| Height (m) | | 1.73 ±0.08 | 1.73 ±0.08 | | | 1.74 ± 0.08 | 0.379^¥^ | | |
| Mean ±SD BMI in kg/m^2^ | | 23.4 ± 3.3 | 23.3 ± 3.0 | | | 24.1 ± 4.5 | 0.143^¥^ | |  |
| *BMI Category (kg/m^2^)* | |  |  | | |  |  |  |  |
| Underweight (<18.5) | | 4 (1.9) | 2 (1.1) | | | 2 (4.9) | 0.007^†^* |  |  |
| Normal (≥18.5 - <25) | | 148 (69.2) | 124 (71.7) | | | 24 (58.5) |  |  |  |
| Overweight (25-29.9) | | 51 (23.8) | 42 (24.3) | | | 9 (22.0) |  |  |  |
| Obesity( ≥30) | | 11 (5.1) | 5 (2.9) | | | 6 (14.6) |  |  |  |
| *Smoking status* | | | | | | | |  |  |
| Current smoker | 137 (64.0) | | 115 (66.5) | | 22 (53.6) | | 0.153^†^ |  |  |
| Former smoker | 21 (9.8) | | 14 (8.1) | | 7 (17.1) | |  |  |  |
| None smoker | 56 (26.2) | | 44 (25.4) | | 12 (29.3) | |  |  |  |
| *Respiratory symptoms*  Yes  No | 153 (71.5)  61 (28.5) | | 125 (72.3)  48 (27.7) | | 28 (68.3)  13 (31.7) | | 0.503^†^ |  |  |
| *Frequency of respiratory symptoms* | | | | | | | |  |  |
| 1-2 per year | 121 (79.1) | | 98 (78.4) | 23 (82.1) | | | 0.660^†^ |  |  |
| 3-6 per year | 14 (9.1) | | 13 (10.4) | 1 (2.6) | | |  |  |  |
| >6 per year | 18 (11.8) | | 14 (11.2) | 4 (14.3) | | |  |  |  |

**Footnote: *Statistical difference: ^†^Chi-square or Fischer’s exact test; ^¥^t-statistics); IQR=Interquartile range; BMI=Body Mass Index; SD=Standard deviation**

**IMPALA respiratory symptom questionnaire**

| **Prompt** | **Label** | **Answer the questions** |
| --- | --- | --- |
| type | name |  |
| start | start | - |
| end | end | - |
| Device id | Device id | - |
| geopoint | geopoint | - |
| Text | Participant ID | Participant ID |
| select_one | sex | Sex |
| date | dob | Date of birth |
| calculate | age_years | Age (years) |
| begin_group | group_respiratory | Symptoms |
| begin_group | group_cough | Cough |
| note | note_respiratory | Questions from BOLD |
| select_one yes_no | cough | Do you usually cough when you don’t have a cold? |
| select_one yes_no | cough_chronic | Are there months in which you cough on most days? |
| select_one yes_no | cough_period | Do you cough on most days for as much as three months each year? |
| integer | cough_years | For how many years have you had this cough? |
| select_one yes_no | sputum | Do you usually bring up phlegm from your chest, or do you usually have phlegm in your chest that is difficult to bring up when you don't have a cold? |
| select_one yes_no | sputum_chronic | Are there months in which you have this phlegm on most days? |
| select_one yes_no | sputum_wet | Is this phlegm frequently wet? |
| select_one yes_no | sputum_positional | Is the phlegm worse when you lie in certain positions (on one side or the other)? |
| select_one yes_no | sputum_period | Do you bring up this phlegm on most days for as much as three months each year? |
| integer | sputum_years | For how many years have you had this phlegm? |
| end_group | group_cough |  |
| begin_group | group_asthma | Wheeze and asthma |
| note | note_asthma | Questions from the Global Asthma Network |
| select_one yes_no | screen_asthma | Have you had wheezing or whistling in the chest in the past 12 months? |
| integer | asthma_attack_n | How many attacks of wheezing have you had in the past 12 months? |
| integer | asthma_attack_sleep_n | In the past 12 months, how many times has your sleep been disturbed due to wheezing? |
| select_one yes_no | asthma_attack_speech | In the past 12 months, has wheezing ever been severe enough to limit your speech to only one or two words at a time between breaths? |
| calculate | asthma_severe | Asthma severity |
| end_group | group_asthma |  |
| begin_group | group_dyspnoea | Breathlessness |
| note | note_dyspnoea | Questions from BOLD, MRC |
| select_one yes_no | walk_disability | Are you unable to walk due to a condition other than shortness of breath? |
| text | walk_disability_info | Please describe the condition |
| select_one yes_no | mrc_5 | Are you too short of breath to leave the room, or short of breath on dressing or undressing? |
| select_one yes_no | mrc_4 | Do you ever have to stop for breath after walking about 100 metres (or after a few minutes) on level ground? |
| select_one yes_no | mrc_3 | Do you ever have to stop for breath after walking at your own pace on level ground? |
| select_one yes_no | mrc_2 | Do you have to walk slower than people of your age on level ground because of shortness of breath? |
| select_one yes_no | mrc_1 | Are you troubled by shortness of breath when hurrying on the level or walking up a slight hill? |
| calculate | mrc_dyspnoea_score | MRC dyspnoea score |
| end_group | group_dyspnoea |  |
| begin_group | group_other | Other symptoms |
| select_one list_weight_loss | weight_loss | In the last 6 months, has your weight changed? |
| select_one yes_no | night_sweats | Have you in the last 4 weeks suffered with night sweats (more than usual for the heat)? |
| end_group | group_other |  |
| begin_group | group_diagnoses | Previous diagnosis |
| note | note_diagnoses | Questions from BOLD and others |
| select_multiple list_copd | copd | Has a doctor or other health care provider ever told you suffer from any of the following? (select all that apply) |
| select_one list_risk_diagnoses | risk_diagnoses | Have you ever suffered from any of the following? (select all that apply) |
| end_group | group_diagnoses |  |
| end_group | group_respiratory | Tuberculosis, Asthma, Bronchitis, Emphysema etc. |

**IMPALA life exposure questionnaire**

| type | name | label::English |
| --- | --- | --- |
| start | start | - |
| end | end | - |
| deviceid | deviceid | - |
| geopoint | geopoint | - |
| text | participantID | Participant ID |
| begin_group | group_home | Exposures at home |
| note | note_home | This section relates to exposures in your home |
| select_one yes_no | smoker_passive_home | Does anyone smoke cigarettes or tobacco inside the building where you sleep (do not include yourself)? |
| select_one yes_no | smoker_passive_work | Does anyone smoke cigarettes or tobacco inside any building where you work or spend other time? |
| begin_group | group_everyday | Daily living |
| note | note_everyday | This section is about exposures from daily living |
| select_one yes_no | screen_non_occupation | In your everyday life, do you breathe in vapours, dusts, gases or fumes for more than 15 hours per week? (not including time you spend in your job) |
| select_one yes_no | refuse_burn | Are you ever exposed to smoke from burning refuse (waste and rubbish)? |
| select_one list_freq | refuse_burn_freq | How often is there smoke from burning refuse where you are? |
| select_one yes_no | aerosol | Do you use aerosols or sprays at home (insecticide, deodorant, cleaning spray and any others) |
| select_one list_freq | aerosol_freq | How often do you use aerosols or sprays at home? |
